# Urine Exosomal AMACR Is a Novel Biomarker for Prostate Cancer Detection at Initial Biopsy

**DOI:** 10.3389/fonc.2022.904315

**Published:** 2022-06-20

**Authors:** Xin Jin, Jin Ji, Decao Niu, Yuchen Yang, Shuchun Tao, Lilin Wan, Bin Xu, Shuqiu Chen, Fubo Wang, Ming Chen

**Affiliations:** ^1^ Department of Urology, Zhongda Hospital, Southeast University, Nanjing, China; ^2^ Surgical Research Center, Institute of Urology, Southeast University Medical School, Nanjing, China; ^3^ Department of Urology, Taizhou People’s Hospital, Taizhou, China; ^4^ Department of Urology, Changhai Hospital, Second Military Medical University, Shanghai, China; ^5^ Center for Genomic and Personalized Medicine, Guangxi Key Laboratory for Genomic and Personalized Medicine, Guangxi Collaborative Innovation Center for Genomic and Personalized Medicine, Guangxi Medical University, Nanning, China; ^6^ Department of Urology, Guangdong Second Provincial General Hospital, Guangzhou, China; ^7^ Nursing Department, Naval Hospital of Eastern Theater Command of People's Liberation Army of China (PLA), Zhoushan, China

**Keywords:** urine exosomes, AMACR, prostate cancer, diagnosis, biomarker

## Abstract

**Objectives:**

The aim of this study is to identify and validate urine exosomal AMACR (UE-A) as a novel biomarker to improve the detection of prostate cancer (PCa) and clinically significant PCa (Gleason score ≥ 7) at initial prostate biopsy.

**Methods:**

A total of 289 first-catch urine samples after the digital rectal exam (DRE) were collected from patients who underwent prostatic biopsy, and 17 patients were excluded due to incomplete clinical information. Urine exosomes were purified, and urinary exosomal AMACR (UE-A) was measured by enzyme-linked immunosorbent assay (ELISA). The diagnostic performance of UE-A was evaluated by receiver operating characteristic (ROC) analysis, decision curve analysis (DCA), and waterfall plots.

**Results:**

The expression of AMACR in PCa and csPCa was significantly higher than that in BPH and non-aggressive (*p* < 0.001). The UE-A presented good performance in distinguishing PCa from BPH or BPH plus non-significant PCa (nsPCa) from csPCa with an area under the ROC curve (AUC) of 0.832 and 0.78, respectively. The performance of UE-A was further validated in a multi-center cohort of patients with an AUC of 0.800 for detecting PCa and 0.749 for detecting csPCa. The clinical utility assessed by DCA showed that the benefit of patients using UE-A was superior to PSA, f/t PSA, and PSAD in both the training cohort and the validation cohort in terms of all threshold probabilities. Setting 95% sensitivity as the cutoff value, UE-A could avoid 27.57% of unnecessary biopsies, with only 4 (1.47%) csPCa patients missed.

**Conclusions:**

We demonstrated the great performance of UE-A for the early diagnosis of PCa and csPCa. UE-A could be a novel non-invasive diagnostic biomarker to improve the detection of PCa and csPCa.

## Introduction

Prostate cancer (PCa) is a serious threat to men’s health. It is one of the leading causes of death in men worldwide. Statistical reports showed that there would be 1,414,259 new cases and 375,304 new deaths in 2020 globally (1) and 268,490 new cases and 34,500 new deaths in the USA (2). Early PCa was confined to the capsule. However, if the tumor invaded the capsule or had metastasis, the treatment was brutal, and the prognosis was poor (2, 3). Therefore, the early diagnosis of PCa has a significant clinical and social value. Prostate-specific antigen (PSA), as the most recognized and most commonly used biomarker of PCa, plays a significant role in detecting PCa at present, but it also has great limitations. A systematic review of PSA screening showed that PSA screening did not clearly reduce cancer mortality (4) but led to overdiagnosis. Another well-known systematic review also published similar results in the same year, showing that PSA reduced cancer mortality but did not improve overall survival and resulted in short-term and long-term surgical complications (5). These studies indicate that PSA as a molecular marker of PCa has low specificity. It is urgent to explore new methods and technologies for early screening and diagnosis of PCa in clinical practice to improve the predictive efficacy of PCa.

Urine is absolutely non-invasive and easy to obtain. The PCa-derived secreted body likely exists in the urine after prostate massage. Protein markers are characterized by solid stability and high specificity. Detection of urine protein after prostate massage will help improve the specificity of PCa diagnosis. Exosomes are small vesicles actively released by cells into the extracellular environment, carrying numerous biomolecules and providing an encouraging non-invasive approach for detecting cancers (6, 7). Recent reports showed that circulating exosomal RNAs could serve as promising biomarkers for cancer detection (6–9). However, urinary exosomal proteins have not been adequately explored as an easily collected and non-invasive source of cancer biomarkers (10).

AMACR (a-Methylacyl-CoA racemase, also known as P504S) is an enzyme that interconverts pristanoyl-CoA and C27-bile acyl CoA between their (R)- and (S)-stereoisomers (11). This protein is elevated in PCa tissue and can act as a biomarker of PCa (12). Urinary and circulating AMACR mRNA has been reported to perform well in PCa diagnosis (13–15). Here, we first evaluated the protein levels of AMACR in urine exosomes between PCa and BPH participants and evaluated its diagnostic performance in differentiating PCa from BPH or csPCa from BPH plus nsPCa patients. We then validated the clinical utility of urine exosomal AMACR to detect PCa and csPCa at initial biopsy.

## Materials and Methods

### Participants

The research was authorized following the Hospital Ethics Committee’s manual by Shanghai Changhai Hospital, Taizhou People’s Hospital and Zhongda Hospital (No. CHEC2013-115). Three sites shared the same standard operating procedure (SOP) for participant recruitment and sample processing. Written informed consents were obtained from the participants before sampling.

A total of 289 consecutive PCa and biopsy-negative control patients with elevated PSA before biopsy were admitted from Changhai Hospital, Taizhou People’s Hospital and Zhongda Hospital, who underwent a prostate biopsy between February 2017 and March 2018. All subjects in this study underwent transperineal biopsy guided by transrectal ultrasound, including patients with PSA > 4 ng/ml and no increase in PSA but with abnormal DRE or imaging examination. The specimen was examined individually by two pathologists and assigned a Gleason score. Baseline information of the study subjects is provided in **Table 1**. Individuals combined with other known tumor histories were excluded. All recruited research subjects signed an informed consent form at admission. We defined benign disease and Gleason score = 6 as the non-aggressive disease and Gleason score ≥7 as the clinically significant PCa (csPCa), which was the same as described in the previous study.

**Table 1:** Demographics and clinical characteristics of participants.

Parameter	Training set				*p*-value	Validation set			*p*-value
	Entire	Negative	Positive			Entire	Negative	Positive	
Age, yr					0.019^*^				0.017^*^
No. pts (%)	139 (100.0)	94 (67.7)	45 (32.3)			133 (100.0)	91 (68.4)	42 (31.6)	
Mean	65.1	64.0	67.4			64.7	63.3	67.6	
SD	7.2	6.9	7.4			8.1	8.4	6.5	
tPSA, ng/ml					0.006^#^				0.53^#^
No. pts (%)	139 (100.0)	94 (67.7)	45 (32.3)			133 (100.0)	91 (68.4)	42 (31.6)	
Median	8.8	8.4	11.0			9.3	9.5	9.2	
IQR	6.6–12.4	6.5–11.1	7.6–13.9			6.9–12.9	6.9–12.6	7.4–13.1	
BMI, kg/m^2^					0.41^#^				0.76^#^
No. pts (%)	139 (100.0)	94 (67.7)	45 (32.3)			133 (100.0)	91 (68.4)	42 (31.6)	
Median	24.2	24.2	24.6			24.2	24.2	24.6	
IQR	22.1–26.5	22.2–26.1	21.9–27.7			22.6–26.2	22.8–26.2	21.9–26.9	
%fPSA					0.002^#^				0.048^#^
No. pts (%)	98 (70.5)	63 (45.3)	35 (25.2)			86 (64.7)	56 (42.1)	30 (22.6)	
Median	0.16	0.19	0.11			0.12	0.14	0.1	
IQR	0.11–0.25	0.12–0.27	0.08–0.18			0.08–0.19	0.08–0.22	0.07–0.13	
PSAD									
No. pts (%)	139 (100.0)	94 (67.7)	45 (32.3)			133 (100)	91 (68.4)	42 (31.6)	
Median	0.18	0.158	0.239			0.18	0.16	0.23	
IQR	0.11–0.25	0.10–0. 25	0.16–0.32			0.11–0.29	0.11–0.25	0.16–0.34	
Biopsy Gleason sum, no. (%)									
6			14 (10.1)					11 (8.3)	
7			16 (11.5)					18 (13.5)	
≥8			13 (9.4)					12 (9)	

yr, years; tPSA, total prostate-specific antigen; SD, standard deviation; IQR, interquartile range; f/t PSA, free prostate-specific antigen/total prostate-specific antigen; f/t PSA, free prostate-specific antigen/total prostate-specific antigen. ^*^Student’s t-test. ^#^Mann–Whitney U test.

### Sample Collection and Preparation

We collected first-catch urine samples following an attentive DRE on the day of the biopsy. The urine samples were processed within 2 h of collection (13). Patients who underwent biopsy were assessed to have an abnormal PSA value (>4 ng/ml), or DRE revealed a nodule or PI-RADS > 3. The diagnosis results were confirmed by biopsy. The pathology diagnosis was double-blind confirmed by two pathologists.

### Exosome Extraction

Exosomes were extracted by a commercial kit according to the manufacturer’s manuals, as in our previous studies (16). In brief, samples were kept on ice and then 20 ml of which was centrifuged at 3,000 *g* at 4°C for 15 min. Afterward, the supernatant was transferred to a centrifuge tube. Reagent A (7.5 ml) was mixed thoroughly with 670 ml of Reagent B. Incubation was conducted at 4°C for 12–16 h, followed by centrifugation at 3,000 *g* at 4°C for 60 min. More than 1 ml of the supernatant was retained, with the residual supernatant discarded. One milliliter of solution was added above the pellet to fully resuspend it. After centrifuging at 10,000 *g* at 4°C for 10 min, the supernatants were discarded. We resuspended the pellet in 200 μl of filtered PBS and then centrifuged it again for 5 min at 10,000 *g* at 4°C. Exosomes were collected in the supernatant and stored in a −80°C refrigerator.

### Transmission Electron Microscopy

Exosome samples were resuspended in PBS. The sample was prepared first and then loaded onto the copper grid. After standing for approximately 20 min at room temperature, the filter paper was used to assimilate excess moisture. The sample was negatively stained for 5 s with 20 μl of 2% phosphotungstic acid (pH 5.52). Then, excess liquid was sucked up by filter paper from the side. After drying at room temperature, the typical structure of exosomes was observed by TEM.

### Western Blot Analysis

The exosome suspension was thawed on ice, and more than 20 μl of RIPA buffer (89901, Thermo Fisher) containing protease inhibitor (B14001, Bimake) was used to lyse for 30 min. The sample was centrifuged at 12,000 *g* for 5 min at 4°C. Then, a BCA kit was used to measure the protein concentration. Then, a 5× loading buffer was added to the supernatant, and the mixture was heated in a metal bath at 97°C for 3 min. Based on the manufacturer’s instructions, a PAGE Gel Fast Preparation Kit (PG112, EpiZyme) was prepared. According to the determined concentration, more than 20–40 μg of protein and protein marker (1610374, Bio-Rad) were each added to the well; SDS-PAGE electrophoresis was conducted at 100 V and run for 105 min. After that, the instrument was set to 100 V and 90 min for electrotransfer. After the transfer was completed, the membranes containing the protein of interest were placed in 5% BAS and blocked for 2 h at room temperature. Then, these were transferred to a primary antibody [including CD9, CD63, CD81, TSG101, Cytochrome c, calnexin (AP1482, Abgent), and ACTB (A5441, Sigma)] and placed on a shaker at 4°C overnight. The next day, the membranes were washed 3 × 5 min by TBST. Incubation of the secondary antibody with the membrane was performed for 2 h at room temperature, followed by three rewashes. Finally, the membrane is photographed.

### Nanoparticle Tracking Analysis

Samples were diluted 1:300 in filtered PBS to control concentrations within the most accurate detection range. Panalytical NanoSight NS300 (Malvern) was used to detect the size and distribution of exosomes. The instrument detection cell was washed with DPBS solution without any nanoparticles. After cleaning the detection module, the diluted sample was added to the syringe and placed on the motorized pump. The testing module should be connected and the manufacturer’s instructions should be followed before testing. To reduce errors, particle diameters were calculated using the Stokes–Einstein equation with three replicates per sample.

### ELISA

Experiments were performed using ELISA kits (E0993h) from EIAab. After the reagents and samples were taken out of the refrigerator, they should be left at room temperature for 30 min, and the working solution should be configured according to the manufacturer’s instructions. The standard was diluted in equal proportions, and 100 μl of the standard or sample was added to the 96-well plate pre-incubated with the primary antibody. Three replicate wells were made for each sample. After blocking with film, the 96-well plate was incubated in a 37°C incubator for 2 h. The liquid in the 96-well plate was discarded, and 100 μl of Reagent A was added. After gentle shaking and placement in a 37°C incubator for 1 h, the liquid was discarded and washed three times with washing working solution (300 μl/well) for 2 min each time. Reagent B operation was similar to a previous operation. Finally, more than 90 μl of the reaction solution was added to each well and then placed in a 37°C incubator for 10–20 min in the dark. When a clear color gradient appeared in the standard wells, more than 50 μl of stop solution was added, and the absorbance value at 450 nm was measured on the computer within 15 min.

### Data Analysis

Data analysis was performed by MedCalc v13.0 (MedCalc Software bvba) and Prism V9.2 (GraphPad software). The age of the different groups was compared by Student’s *t*-test. The Mann–Whitney *U* test was used to compare PSAD, PSA, and f/t PSA by the nonparametric test. Pearson’s chi-squared test compared DRE status. Exosomal AMACR was compared in different groups using a nonparametric test. A univariate logistic regression was used to identify independent predictors of PCa based on biopsy results. We evaluated the diagnostic value of the parameters using the receiver operating characteristic (ROC) curve and the area under the curve (AUC). Comparisons of AUC between different indicators were made using MedCalc and Delong methods. Patients’ net benefit was evaluated using decision curve analysis (DCA). Two-sided *p*-values were used, and *p* < 0.05 was considered statistically significant.

## Results

### Participants’ Characteristics

The baseline information of the training and validation cohorts of patients is shown in **Table 1**. We collected the urine sample of 289 patients from February 2017 to March 2018 and excluded 17 patients due to incomplete clinical information. Finally, we analyzed 272 patients (including 185 controls and 87 PCa patients, with a positive biopsy rate of 31.98%). The mean age of participants in the training and validation cohorts was 65.1 years (SD 7.2) and 64.7 years (SD 8.1), respectively. Not all patients underwent MRI. Sixty-six (47.5%) patients in the training cohort and 58 (43.6%) patients in the validation cohort had MRI results. The median tPSA of participants in training and validation cohorts were 8.8 (IQR: 6.6–12.4) and 9.3 (IQR: 6.9–12.9), respectively. For the f/t PSA, the medians were 0.16 (IQR: 0.11–0.25) in the training cohort and 0.12 (IQR: 0.08–0.19) in the validation cohort. The median PSAD was 0.18 (IQR: 0.11–0.25) in the training cohort and 0.18 (IQR: 0.11–0.29) in the validation cohort. The results of exosome identification refer to the literature previously published by our group (16).

### Urine Exosomal AMACR Could Distinguishing PCa From BPH and BPH Plus Non-Aggressive PCa

**Figure 1:**
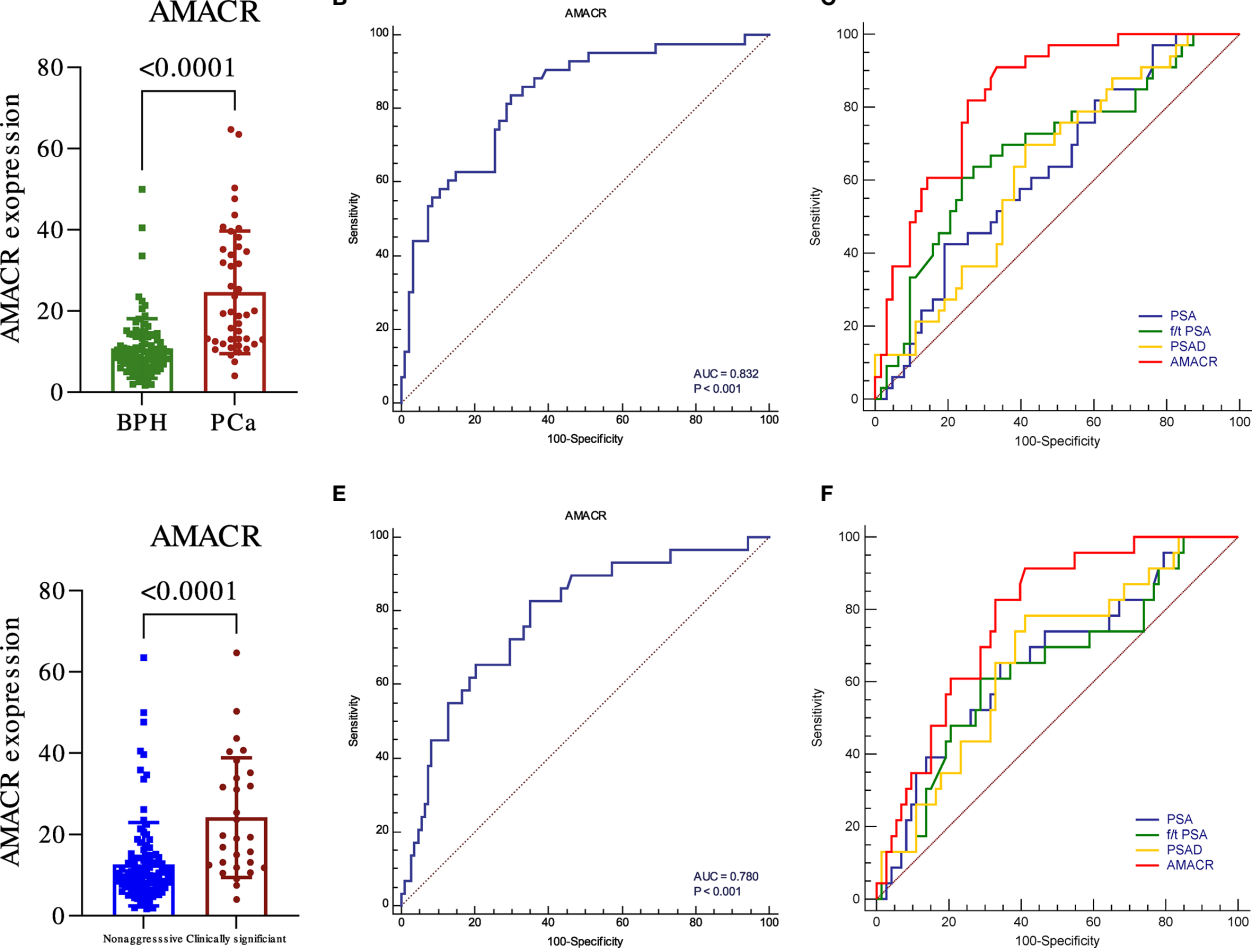
The diagnostic utility of urine exosomal AMACR in the training cohort. The urine AMACR was significantly higher in the PCa **(A)** (p < 0.001) and csPCA **(D)** (p < 0.001) than in the control group. The utility of urine AMACR in distinguishing PCa **(B)** (AUC: 0.832, p < 0.001) and csPCa **(E)** (AUC: 0.780, p < 0.001). Comparison ROC illustrated that the urine AMACR has a better performance than PSA, f/t PSA, and PSAD in PCa **(C)** and csPCa **(F)** diagnosis.

The expression of AMACR in PCa and csPCa was significantly higher than that in BPH and non-aggressive (**Figures 1A, D**, *p* < 0.001). The diagnostic performance of AMACR, evaluated by ROC, was 0.832 for detecting PCa from BPH (**Figure 1B**, *p* < 0.001) and 0.78 for detecting clinically significant PCa (csPCa) (**Figure 1E**, *p* < 0.001) from BPH plus non-aggressive PCa. AMACR was superior to PSA, PSAD, and f/t PSA in detecting PCa from BPH (**Figure 1C**, AMACR vs. PSA, *p* = 0.0054; AMACR vs. f/t PSA, *p* = 0.056, AMACR vs. PSAD, *p* = 0.008). Diagnostic efficiency (*p* = 0.0054), and compared with f/t PSA, the *p*-value was 0.056. For the diagnosis of csPCa, AMACR was also superior to PSA, f/t PSA, and PSAD but could not reach statistical significance (**Figure 1F**, AMACR vs. PSA, *p* = 0.1838; AMACR vs. f/t PSA, *p* = 0.125, AMACR vs. PSAD, *p* = 0.214). The detailed information is summarized in **Table 2**.

**Table 2:** The performance of urine exosomal AMACR and clinical features to predict biopsy results in the training cohort.

Parameters	Positive and negative		Non-aggressive and csPCa	
	AUC (95% CI)	Univariate *p*	AUC (95% CI)	Univariate *p*
Age	0.622 (0.536 to 0.703)	0.008	0.631 (0.546 to 0.712)	0.014
BMI	0.544 (0.458 to 0.629)	0.746	0.541 (0.454 to 0.625)	0.211
PSA	0.645 (0.559 to 0.724)	0.017	0.674 (0.589 to 0.751)	0.006
f/t PSA	0.689 (0.588 to 0.779)	0.002	0.645 (0.542 to 0.740)	0.020
PSAD	0.692 (0.608 to 0.767)	<0.001	0.700 (0.616 to 0.774)	<0.001
AMACR	0.832 (0.759 to 0.890)	<0.001	0.780 (0.701 to 0.846)	<0.001

AUC, area under the curve; PSA, prostate-specific antigen; PSAD, prostate-specific antigen density; f/t PSA, free prostate-specific antigen/total prostate-specific antigen; csPCa, clinically significant prostate cancer.

### The Diagnostic Performance of Urine Exosomal AMACR Was Validated in an Additional Cohort of Participants

**Figure 2:**
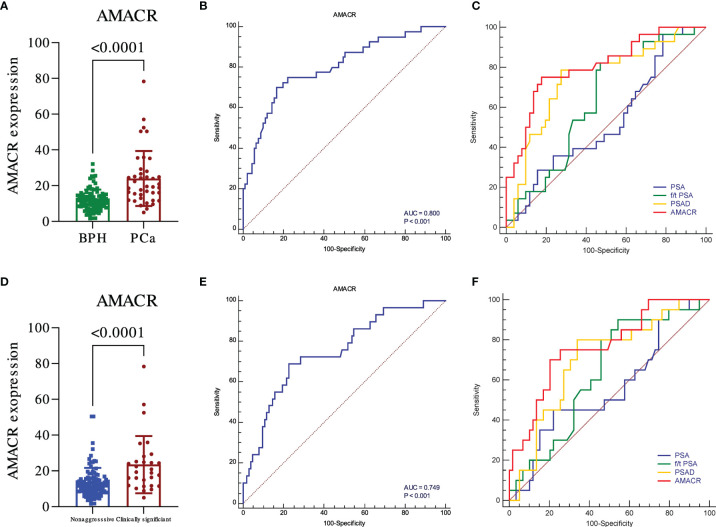
The diagnostic utility of urine exosomal AMACR in the validation cohort. The urine AMACR was significantly higher in the PCa **(A)** (p < 0.001) and csPCA **(D)** (p < 0.001) than the control group, respectively. The utility of urine AMACR in distinguishing PCa **(B)** (AUC: 0.800, p < 0.001) and csPCa **(E)** (AUC: 0.749, p < 0.001). Comparison ROC illustrated that the urine AMACR has a better performance than PSA, f/t PSA, and PSAD in PCa **(C)** and csPCa **(F)** diagnosis.

We further evaluated the levels of urine AMACR in the samples of the validation cohort of participants. Similar results were observed. The expression of AMACR was upregulated in PCa compared to BPH (**Figure 2A**, *p* < 0.001) and also significantly upregulated in PCa compared to BPH plus non-aggressive PCa (**Figure 2D**, *p* < 0.001). The ROC was 0.800 for detecting PCa from BPH (**Figure 2B**, *p* < 0.001) and 0.749 for detecting clinically significant PCa (csPCa) from BPH plus non-aggressive PCa (**Figure 2E**, *p* < 0.001). Compared to clinical parameters, AMACR was superior to PSA, f/t PSA (**Figure 2C**, AMACR vs. PSA, *p* = 0.001; AMACR vs. f/t PSA, *p* = 0.032), and PSAD but could not reach statistical significance (**Figure 2C**, AMACR vs. PSAD, *p* = 0.06) in detecting PCa from BPH. For the diagnosis of csPCa, AMACR was also superior to PSA (**Figure 2F**, AMACR vs. PSA, *p* = 0.031), f/t PSA, and PSAD but could not reach statistical significance (**Figure 2F**, AMACR vs. f/t PSA, *p* = 0.109, AMACR vs. PSAD, *p* = 0.115). The detailed information is summarized in **Table 3**.

**Table 3:** The performance of urine exosomal AMACR and clinical features to predict biopsy results in the validation cohort.

Parameters	Positive and negative		Non-aggressive and csPCa	
	AUC (95%CI)	Univariate *p*	AUC (95%CI)	Univariate *p*
Age	0.629 (0.541 to 0.711)	0.002	0.641 (0.553 to 0.722)	0.003
BMI	0.517 (0.428 to 0.604)	0.984	0.534 (0.445 to 0.621)	0.989
PSA	0.534 (0.446 to 0.621)	0.549	0.580 (0.491 to 0.665)	0.211
f/t PSA	0.634 (0.520 to 0.738)	0.044	0.634 (0.520 to 0.738)	0.133
PSAD	0.664 (0.577 to 0.744)	0.015	0.669 (0.582 to 0.748)	0.030
AMACR	0.800 (0.721 to 0.865)	<0.001	0.749 (0.666 to 0.821)	<0.001

AUC, area under the curve; PSA, prostate-specific antigen; f/t PSA, free prostate-specific antigen/total prostate-specific antigen; csPCa, clinically significant prostate cancer; PSAD, prostate-specific antigen density.

### The Clinical Application of Urine Exosomal AMACR

**Figure 3:**
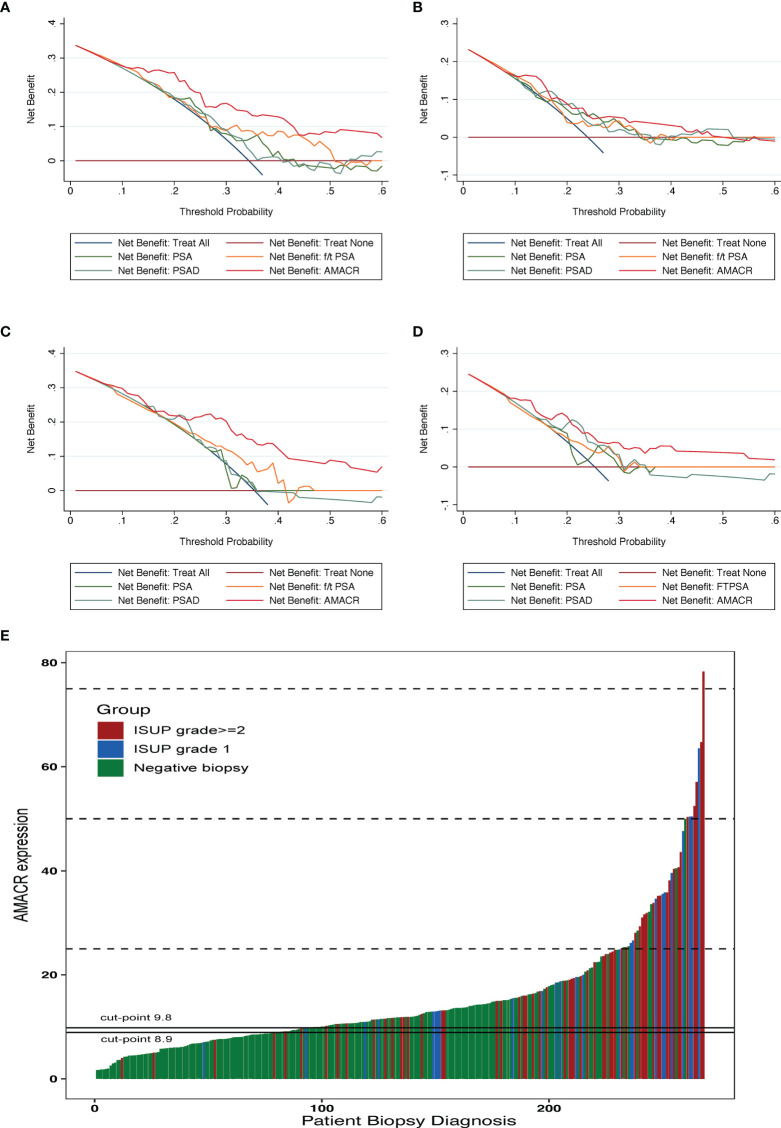
Clinical application of the urine exosomal AMACR. The DCA indicates that urine AMACR has a higher net benefit across a threshold of 20%–50% probabilities for diagnosing PCa **(A, C)** and csPCa **(B, D)** in two cohorts. **(E)** Waterfall plot of the urine AMACR in relation to prostate biopsy results (*n* = 272). Red bar indicates the ISUP grade ≥2 tumors (GS ≥ 7); the blue one indicates the ISUP grade 1 tumors (GS = 6); the green one indicates the negative biopsies. Two horizontal lines represent the cutoff points of 9.8 at a sensitivity of 90% and 8.9 at a sensitivity of 95%.

To determine the net benefit of participants, the clinical DCA was used (**Figures 3A–D**). The results showed that the benefit of patients using AMACR was superior to PSA, f/t PSA, and PSAD in both the training cohort and the validation cohort in terms of diagnostic efficiency. There are also good performances in diagnosing csPCA. Setting 30% as the threshold, AMACR could avoid 60.4% of unnecessary biopsies in the training cohort, which was significantly higher than 47.4% in PSA, with only 12 (8.6%) csPCA patients missed. AMACR could avoid 58.6% of unnecessary biopsies in the validation cohort, which was significantly higher than 26.3% in PSA, with only 10 (7.5%) patients missed, among which 9 (6.8%) of these patients had clinically significant PCa. Setting 95% sensitivity as the cutoff value 8.9, UE-A could avoid 27.57% of unnecessary biopsies, which was significantly higher than 13.24% in PSA, with only 4 (1.47%) csPCa patients missed. The waterfall plot shows the biopsy results of each participant and AMACR results (**Figure 3E**).

## Discussion

The diagnostic rate of PCa has steadily increased with the increased PSA screening (17)... In spite of abnormal DRE and transrectal ultrasound (TRUS) triggering prostate biopsy, PSA is still the main indicator for prostate biopsy (18). Higher levels of PSA are routinely used in predicting PCa risk. Scientists are therefore encouraged to develop more specific biomarkers for detecting clinically significant PCa due to the low specificity of PSA testing for screening PCa and its limitations in identifying clinically significant PCa at an early stage and avoiding unnecessary biopsies (19).

AMACR is a racemase encoded by the P504S gene, which plays a vital role in the β oxidation of fatty acids and cholic acid metabolism (20). Lee et al. reported that AMACR has a diagnostic and prognostic value in glioblastoma (21). In PCa, AMACR can cause DNA damage that leads to the expression of peroxide, which promotes tumor progression (12). At present, many studies have pointed out that the AMACR mRNA level is significantly highly expressed in PCa tissues, and its sensitivity and specificity as a diagnostic marker are 82%–100% and 97%–100%, respectively (22, 23). Rogers et al. (24) first proposed the potential of AMACR as a diagnostic marker for PCa in the current study on the diagnostic value of AMACR protein level in urine. After testing the urine samples of 26 patients with PCa biopsy, they found that the sensitivity of AMACR for diagnosis was 100%, but the specificity was only 58%. This may be due to the limitation of sample size. However, in the study of Sroka et al. (25), AMACR level in the PCa group was higher than the control group (*p* < 0.001), but its AUC as a diagnostic indicator was 0.748, slightly lower than the diagnostic efficacy of serum PSA (AUC = 0.769) and could not replace PSA as a new diagnostic marker. Here, we showed that urine exosomal AMACR achieved an AUC of 0.832 in detecting PCa from BPH and an AUC of 0.78 in predicting csPCa at initial biopsy. Moreover, the diagnostic performance of urine exosomal AMACR was superior to PSA, f/t PSA, and PSAD. The similar results were observed in an additional cohort of patients. These results indicated that urine exosomal AMACR could serve as a promising biomarker to improve the detection of PCa and csPCa.

Research conducted in the present study may provide a new perspective on PCa and csPCa diagnosis. Several limitations remain, however. First, the sample size was inadequate. We need another multi-center, perspective, large-scale study to verify our findings. Second, we did not compare UE-A to other emerging assays, such as MiPS, SelectMDx, and EPI. Third, UE-A was significantly correlated with PI-RADS (**Figure S1**, *p* = 0.0020). However, we could not compare the performance of UE-A to that of PI-RADS because more than half of patients did not undergo MRI. Finally, given the noticeable differences in genetic alteration signatures between Asians and Westerners (26), additional studies should be conducted to compare the clinical utility of our UE-A in Asian and Western patients.

## Conclusion

In summary, we developed and validated a new non-invasive, urinary-based, exosomal biomarker, AMACR, for the detection of PCa and csPCa early in the disease course. Clinically, the urine exosomal AMACR had a higher net benefit than current clinical parameters, while it could spare a significant amount of unnecessary biopsies.

## Data Availability Statement

The raw data supporting the conclusions of this article will be made available by the authors, without undue reservation.

## Ethics Statement

The studies involving human participants were reviewed and approved by the Ethics Committee of Shanghai Changhai Hospital, the Ethics Committee of Taizhou People’s Hospital, and the Ethics Committee of Zhongda Hospital. The patients/participants provided their written informed consent to participate in this study.

## Author Contributions

MC and FW led the study. XJ, JJ, and DN contributed to the conception of the study. XJ, JJ, DN, YY, ST, and BX performed the experiment. JJ, XJ, DN, SC, and YY contributed significantly to analysis and manuscript preparation. FW and JJ performed the data analyses and wrote the manuscript. XJ, DN, and ST helped perform the analysis with constructive discussions. FW and MC critically reviewed the manuscript. All authors contributed to the article and approved the submitted version.

## Funding

This work was funded by the National Natural Science Foundation of China (NSFC) (81902616 to FW) and the Science and Technology Support Project in the Field of Biomedicine of Shanghai Science and Technology Action Plan (19441909200 to FW).

## Conflict of Interest

The authors declare that the research was conducted in the absence of any commercial or financial relationships that could be construed as a potential conflict of interest.

## Publisher’s Note

All claims expressed in this article are solely those of the authors and do not necessarily represent those of their affiliated organizations, or those of the publisher, the editors and the reviewers. Any product that may be evaluated in this article, or claim that may be made by its manufacturer, is not guaranteed or endorsed by the publisher.
