# Supplementary material for: Urine Exosomal AMACR Is a Novel Biomarker for Prostate Cancer Detection at Initial Biopsy
Source: Front Oncol. 2022 Jun 20;12:904315. doi: 10.3389/fonc.2022.904315 (PMC9251007; doi:10.3389/fonc.2022.904315)
Supplement: Supplementary file 1 [file Image_1.pdf]

Supplementary information

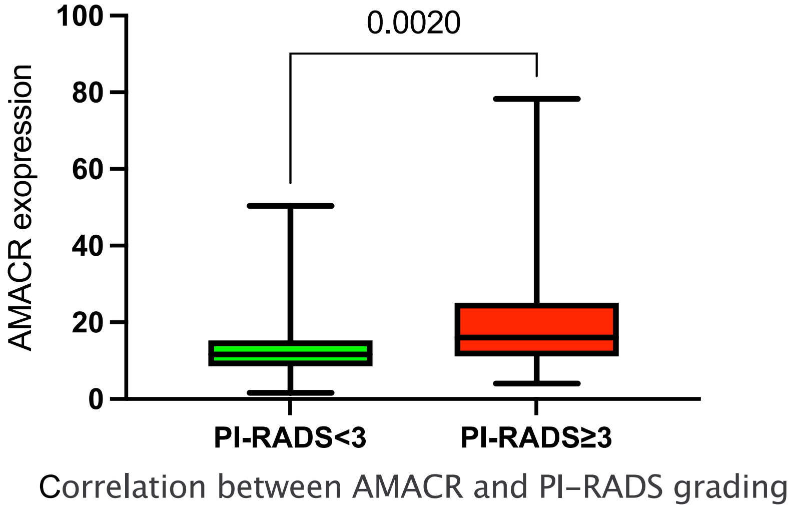

Figure S1 Correlation between AMACR expression and PI-RADS assessment.
